# The PiGeOn project: protocol of a longitudinal study examining psychosocial and ethical issues and outcomes in germline genomic sequencing for cancer

**DOI:** 10.1186/s12885-018-4366-x

**Published:** 2018-04-23

**Authors:** Megan Best, Ainsley J. Newson, Bettina Meiser, Ilona Juraskova, David Goldstein, Kathy Tucker, Mandy L. Ballinger, Dominique Hess, Timothy E. Schlub, Barbara Biesecker, Richard Vines, Kate Vines, David Thomas, Mary-Anne Young, Jacqueline Savard, Chris Jacobs, Phyllis Butow

**Affiliations:** 1 Psycho-oncology Co-operative Research Group (PoCoG), Level 6 North, Lifehouse (C39Z), University of Sydney, Sydney, NSW 2006 Australia; 2 Sydney Health Ethics, Sydney School of Public Health, University of Sydney, Sydney, NSW 2006 Australia; 3 Centre for Medical Psychology and Evidence-based Decision-making, School of Psychology (CeMPED – Psychology), University of Sydney, Sydney, NSW 2006 Australia; 4 Prince of Wales Clinical School, UNSW Sydney, Sydney, NSW 2052 Australia; 5 Hereditary Cancer Centre, Prince of Wales Hospital, Sydney, NSW 2052 Australia; 6 Cancer Division, Garvan Institute of Medical Research, 384 Victoria St, Darlinghurst, NSW 2021 Australia; 7 Sydney School of Public Health, University of Sydney, Sydney, NSW 2006 Australia; 8 National Human Genome Research, National Institutes of Health, 31 Center Drive, MSC 2073, Bethesda, MD 20892 USA; 9 Rare Cancers, PO Box 440, Bowral, NSW 2576 Australia; 10 Genome One, Garvan Institute of Medical Research, 384 Victoria St, Darlinghurst, NSW 2021 Australia

**Keywords:** Genomics, Neoplasm, Psychosocial factors, Ethical issues, Genetic testing, Cancer, Germline genomic sequencing

## Abstract

**Background:**

Advances in genomics offer promise for earlier detection or prevention of cancer, by personalisation of medical care tailored to an individual’s genomic risk status. However genome sequencing can generate an unprecedented volume of results for the patient to process with potential implications for their families and reproductive choices. This paper describes a protocol for a study (PiGeOn) that aims to explore how patients and their blood relatives experience germline genomic sequencing, to help guide the appropriate future implementation of genome sequencing into routine clinical practice.

**Methods:**

We have designed a mixed-methods, prospective, cohort sub-study of a germline genomic sequencing study that targets adults with cancer suggestive of a genetic aetiology. One thousand probands and 2000 of their blood relatives will undergo germline genomic sequencing as part of the parent study in Sydney, Australia between 2016 and 2020. Test results are expected within12–15 months of recruitment. For the PiGeOn sub-study, participants will be invited to complete surveys at baseline, three months and twelve months after baseline using self-administered questionnaires, to assess the experience of long waits for results (despite being informed that results may not be returned) and expectations of receiving them. Subsets of both probands and blood relatives will be purposively sampled and invited to participate in three semi-structured qualitative interviews (at baseline and each follow-up) to triangulate the data. Ethical themes identified in the data will be used to inform critical revisions of normative ethical concepts or frameworks.

**Discussion:**

This will be one of the first studies internationally to follow the psychosocial impact on probands and their blood relatives who undergo germline genome sequencing, over time. Study results will inform ongoing ethical debates on issues such as informed consent for genomic sequencing, and informing participants and their relatives of specific results. The study will also provide important outcome data concerning the psychological impact of prolonged waiting for germline genomic sequencing. These data are needed to ensure that when germline genomic sequencing is introduced into standard clinical settings, ethical concepts are embedded, and patients and their relatives are adequately prepared and supported during and after the testing process.

## Background

Despite substantial therapeutic progress, over one-third of patients diagnosed with cancer will die of their disease [1]. Early risk identification and/or detection of cancer is vital to improving outcomes through prevention and early diagnosis, with the potential to increase the cancer cure rate.

Cancer may be regarded as the product of cumulative somatic genetic mutations, with some arising in individuals with a germline variant conferring an increased risk, modified by environmental exposures. It is estimated that the heritable component of common cancers such as colorectal, breast and prostate cancer contribute to 25–50% of their aetiology, [2] although the majority of this percentage remains to be elucidated [3, 4]. Advances in genomics offer great promise toward improving outcomes in prevention. The introduction of massively parallel sequencing has rapidly reduced the cost and processing time of germline genomic sequencing (GGS), and as a result, an unprecedented amount of both somatic and germline information has been generated [5]. Advances including the ability to test multiple genes in a single panel instead of individual genes, and GGS, have been particularly important in achieving increased access to genomics [6]. These developments have the potential to immediately impact clinical care through production of an exponential increase in information about germline cancer genetics, which in turn may assist in the generation of knowledge to identify those at increased risk [7].

The cost and effectiveness of screening and prevention programs in general is dependent on targeting high-risk populations [8]. Clinical criteria for hereditary cancer based on family history – the most common criteria to inform current practice - do not identify all variant carriers and are unlikely to identify de novo mutations, recessive alleles, or multiplicative effects of polygenic risk [9]. The capacity to define who is at increased risk, and perhaps just as importantly, who is not at increased risk, has both individual and public health implications.

Genomic sequencing to define an individual’s risk may soon offer a universal, acceptable and cost-effective method of personalising medical prevention strategies tailored to genetic status that predicts risk of developing new cancers. This study will focus on GGS. A second study examining the psychosocial and ethical issues and outcomes of tumour genomic profiling for patients with advanced cancer is underway and is described in a separate paper. These two studies comprise the P (psychosocial) I (in) GE (genomic) ON (oncology) Project.

GGS determines the complete DNA sequence of an organism’s genome which in humans, includes ~ 20,000 genes [10]. GGS can reveal germline genetic variants that may also affect blood relatives, that are: i) relevant to the target cancer and clinically actionable, guiding risk prevention; ii) relevant to the target cancer but not clinically actionable, (no proven treatments); iii) incidental (relevant to other cancers and diseases) and clinically actionable, guiding risk prevention; iv) incidental and not clinically actionable (no proven treatments); or v) of unknown or uncertain significance. Depending on a test’s methods and filtering, results of GGS can be highly complex [11].

Individuals with pathogenic germline variants that signal high risk of a particular disease(s) (and their blood relatives) can be offered more intensive risk management. But GGS will only realize its potential in an effective and ethically appropriate way if patients can understand, manage, and make informed decisions to pursue health recommendations based on genomic results. A number of challenges inherent in GGS present barriers to these outcomes.

Although uncertainty pervades medical information, its scope in genomics may be unprecedented [12]. Han et al.’s taxonomy of medical uncertainties in clinical genome sequencing [13] identifies three principal sources of uncertainty: indeterminate outcomes (probability), imprecise risk estimates, and complexity. Given that the science of linking genetic variants to disease risk is in its nascent stage, practitioners who obtain patient consent for GGS face a challenge in communicating these uncertainties to guarantee informed choice and reduce unrealistic expectations. At the same time, patients must absorb and cope with a large amount of information both before sequencing (during the consent process), after sequencing (during the return of results), and at follow up [14].

Patient uncertainty and poor tolerance of ambiguity has been found to reduce patient willingness to receive GGS results [15–17]. While patient autonomy and shared decision-making are recognized values in Australian health care, what information patients want or should be told, and how uncertainty should be approached in GGS, is not well understood [18]. In view of the fact that uncertainty will continue to pervade GGS for years to come, it has been suggested that, rather than seeing uncertainty as something that needs to be eradicated, it should be embraced as a part of the process, with communication in the therapeutic relationship adapted to support the patient appropriately [19]. Debate is ongoing; and represents “arguably the most pressing issue in genetics today” [20].

The psychological impact of single gene testing has been well studied, particularly in the context of hereditary breast and ovarian cancer, Lynch syndrome and melanoma [21]. The evidence suggests that distress lessens or remains stable for those found not to have a pathogenic variant, and while there is often an initial increase in distress for those found to have a gene variant, distress generally returns to normal levels in the longer term [22, 23]. We note that there are important differences between genome sequencing and single gene testing that have implications for psychological outcomes, making it unlikely that single gene results will generalize to whole genome sequencing. First, in GGS, there can be (depending on reporting/filtering methods) an unprecedented volume of results to process. The potential for incidental findings means that both patients and families may be faced with risks they had not been seeking nor were prepared to learn. Second, the high incidence of findings of unknown/uncertain significance, whose meaning may or may not become clearer over time, may be confusing and worrying to patients and families. Third, results may be both diagnostic and predictive, each with traditionally different ethical norms guiding clinical practice [24, 25]. Thus psychosocial and ethical assessment of GGS is critical to guide implementation into mainstream medicine.

There has also been a question raised regarding whether GGS can deliver behavioural change in cancer care, as several studies of allegedly healthy participants have indicated that this was not always the case following receipt of actionable results [26–30]. Most participants in these studies seemed not unduly concerned or distressed about their health at short-term follow-up, and did not change their lifestyle or start more intensive screening. However, it is not clear whether these results could be extrapolated to a cancer context, where patients are more likely to view the disease as very serious and to seek optimal treatment and/or prevention [31]. The vast majority of genetic studies in hereditary cancer families have reported that the majority of participants change their behaviour including undergoing prophylactic surgery [32–34].

Research to date has revealed cautious interest in genomic testing in the general population. Several Australian studies have found limited knowledge about, and little interest in pursuing, direct-to-consumer personal genomic testing amongst the general public, [35] who have also expressed significant concerns about issues such as privacy and potential discrimination [35–38].

Interest among cancer patients appears stronger [39]. A US study [40] exploring hypothetical responses to an offer of GGS, found that many cancer patients were interested in testing, and had more faith in genomic over standard test results. Patients appear to value these tests sufficiently to pay substantial amounts of money ($1000–$2000 for a pharmacogenomic test evaluating likely response to particular drugs) and to wait for their results for up to two weeks [41]. Most patients in this latter study wanted to be involved in decision-making about the test, but one in five lacked a basic understanding of this approach, a result also found in other studies [16, 35]. There is evidence that previous experience of illness and/or family history of disease can impact attitudes [42–45]. However, it is salient to remember that although hypothetical scenarios for Huntington’s Disease implied that 85% at risk would take up mutation testing, in practice only 15% did [46].

Overall, there is insufficient understanding of the preferences, attitudes and values of cancer patients who have actually had GGS: their expectations, their experience of uncertainty, or the psychological effects of testing. The Clinical Sequencing Exploratory Research studies [47] and ClinSeq [48] are currently collecting data in apparently healthy individuals, but no studies of cancer patients who have actually undergone GGS testing have reported longitudinal data. Furthermore, no studies have explored the impact of GGS on blood relatives, yet germline findings have potential implications for blood relatives as for patients. It is important to explore how patients and, blood relatives value and experience GGS, and cope with prolonged uncertainty, non-actionable results and incidental findings, as well as informative findings, before GGS enters routine clinical practice.

This report will outline the first Australian study to collect longitudinal data on cancer patients’ experiences of GGS, and one of the first studies internationally to follow probands and their blood relatives who undergo GGS, over time. The study results will inform ongoing ethical debate and clinical practice development on issues such as protocols for obtaining informed consent for GGS, and informing people and their relatives regarding genomic results. The study will also provide critical outcome data concerning the psychosocial impact of waiting for GGS results on patients and their blood relatives. These data are needed to ensure that when GGS is introduced into routine clinical care, ethical concepts are embedded, and patients and their relatives are adequately prepared and supported during and after the testing process.

### Guiding theory

There is a strong evidence base supporting the use of social cognition models to provide a structured framework for identifying psychosocial and cognitive influences on health behavior including, genetic testing [49]. This study’s design is guided by Protection Motivation Theory [50] and Differentiation and Consolidation theory [51]. Protection Motivation Theory proposes that we defend ourselves according to the apparent severity and probability (vulnerability) of danger, apparent effectiveness of protective behaviour, and apparent self-efficacy in executing the protective behaviour. Differentiation and Consolidation theory asserts that decision-making involves a method of ongoing differentiation including: recognizing options with apparent critical attributes, prioritising one or two possibilities based on highly rated attributes and reassessing an initial preference on the basis of new information. This is followed by a consolidating process, which emphasises one’s values, and future potential outcomes, to favourably support the chosen route and thereby prepare for possible threats, regret and doubt.

In this study, protection motivation will ensue from perceived susceptibility to disease risk, and/or fear of cancer progression, as well as participant and proband knowledge of, attitudes to, and value given to, GGS (as a strategy to guide more effective screening and thus reduce the threat of cancer development). Patients who understand GGS to be valuable will be prone to remain satisfied with their decision to undertake GGS, over time. Intolerance of uncertainty may make GGS less attractive and intensify regret, doubt and poor psychological outcomes.

## Methods/Design

### Aims and hypotheses

The aim of this study in people who undergo GGS is to:

1. evaluate the impact of testing on cancer-related anxiety, patients’ and blood relatives’ perceived value of genomic information, and
2. undertake a critical reflection on all results, with reference to relevant ethical issues and concepts.

### Primary outcomes

1. The primary psychosocial endpoint is: impact of testing on cancer-related distress, measured by change in the Multidimensional Impact of Cancer Risk Assessment (adapted) Scale.
2. The primary attitudinal endpoint is: patients’ and blood relatives’ perceived value of genomic information measured by a hypothetical time trade-off scenario.
3. The primary ethical outcome is: a critical reflection on all results, within reference to a range of normative ethical issues and concepts.

### Secondary outcomes

1. Secondary psychosocial /ethical outcomes include significant changes in scores of any of the following: Impacts of Events Scale Hospital Anxiety and Depression Scale; Fear of Cancer Occurrence/Recurrence/Progression; Herth Hope Scale; risk perception; perceived susceptibility to cancer; and perceived likelihood of having a pathogenic variant.
2. Secondary attitudinal outcomes include: understanding of GGS; views on disclosure of genomic information; perceived benefits and drawbacks of GGS.
3. Secondary decisional outcomes include: degree of decisional satisfaction and regret.

### Parent study

The Genomic Cancer Medicine Program, funded by the NSW Ministry of Health, Australia, involves the parent study, Genetic Cancer Risk in the Young (Cancer Risk) Study. This study is prospectively recruiting 1000 cancer patients with features suggestive of a genetic aetiology. Two thousand first degree blood relatives of the cancer probands will also be recruited. Both probands and relatives will be offered GGS. The Cancer Risk Study participants will receive results 12–15 months after initial enrolment if actionable results are found, and study participants wish to receive them. Risk management will be offered within a clinical trial setting as part of a separate funded study, the Surveillance in Multi-Organ Cancer prone syndromes study. Patients and blood relatives will be recruited to the Cancer Risk psychosocial study when they give written consent to the Cancer Risk Study. Thus they will have already considered and given consent to GGS but not yet had GGS testing.

The current PiGeOn study represents a sub-study of a germline genomic study targeting people with cancers likely to have genetic aetiology.

This study is led by the Psycho-Oncology Co-operative Research Group based at the University of Sydney, Australia. The project was funded by a project grant from the National Health and Medical Research Council, Australia, and ethics approval was given by the Human Research Ethics Committee at St Vincent’s Hospital, Sydney.

### Research design

This study is a mixed method, prospective, cohort sub-study of a GGS program recruiting probands with a history suggestive of a genetic aetiology and two of their first-degree blood relatives.

### Setting

Participants will be recruited by the parent study, from incident and prevalent cases at oncology units in Sydney hospitals. All eligible participants will be asked to give consent using established protocols for genetic research, validated by large genetic studies, the Kathleen Cuningham Foundation Consortium for research into Familial Breast cancer [52] and the International Sarcoma Kindred Study [53]. This consent covers GGS as well as psychosocial questionnaires and interviews. Participants can elect whether and which type of results (i.e. ‘gene variant that causes cancer’, ‘incidental finding that may be important to my health’) they want returned. Reporting of variants of uncertain significance will not be offered.

### Participants

*Inclusion criteria* include having a histologically confirmed malignancy, age 16–40 years at diagnosis, or an individual with > 1 primary cancer diagnosed < 50 yrs. of age or an individual with > 2 primary cancers at any age; first degree relative aged 18 years or over of an individual meeting the above criteria or a cancer affected blood relative aged 18 years or older of an individual meeting the above criteria; willing and able to comply with all study requirements, including timing and/or nature of required assessments; signed, written informed consent to participation in GGS.

*Exclusion criteria* include: inability to understand an English language consent form.

### Procedure for PiGeOn

**Fig. 1:**
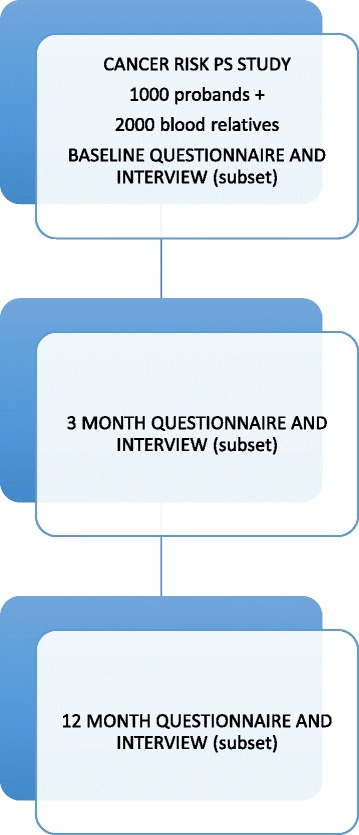
Study Procedure. Cancer risk PS study: Cancer risk psycho-social sub-study

As seen in Fig. 1, participants will be asked to complete a questionnaire at baseline, 3 months after baseline (follow-up 1) and 12 months after baseline (follow-up 2). It is expected that no participant will have received results before follow-up 2. These timeframes were chosen to allow impact of prolonged uncertainty to be explored. A subset of both proband and blood relative groups (approximately 25 participants each) will be asked to participate in semi-structured interviews in parallel with questionnaire time points to further investigate attitudes towards GGS and its psychosocial, and ethical aspects.

### Measures

Patient-reported outcomes will be measured according to the following schedule (see Table 1).

**Table 1:** Survey measures

Domain	Measures	Baseline (T0)	Follow-up 1 (T1) (3 months post-baseline)	Follow-up 2 (T2) (12 months post-baseline)
Demographics				
Age, gender, marital status, education level, occupation etc	Demographic items	x		
Views and attitudes				
Perceived importance of GGS	Adapted from Hay et al. [54]	x		
Knowledge	Study-developed	x		
Preferences for who tested	Ballinger et al. [55]	x		
Value of GGS	Adapted from previous studies [41, 56, 57]	x		
Result return preferences	Ballinger et al. [55]	x		
Perceived benefits and drawbacks of GGS	Adapted from Jamal et al. [61]		x	x
Anticipated behavioural change	Study-developed	x		
Psychological factors				
Coping with uncertainty	Kasparian et al. [60]	x		
Self-efficacy	Adapted from Rosenberg et al. [59]	x		
Perceived susceptibility	Kasparian et al. [60]	x	x	x
Psychological outcomes				
Coping with waiting for GGS results	Multidimensional impact of cancer risk assessment (adapted) [64]		x	x
Fear of cancer (recurrence)	Concerns about Recurrence Questionnaire [67]	x	x	x
Cancer specific anxiety	Impact of events scale [62]		x	x
Anxiety and depression	Hospital anxiety and depression scale [63]		x	x
Hope	Herth Hope Index [65]		x	x
Decisional outcomes				
Decisional satisfaction	Satisfaction with Decision Scale [68]	x	x	x
Decisional regret	Decision Regret Scale [66]		x	x

*GGS* germline genome sequencing

The following outcome measures will be administered at baseline only:

#### Demographic data

Age, gender, marital status, ancestry, language spoken at home, medical history, family history of cancer, lifestyle habits, socio-economic variables and history of genetic screening will be gathered by patient report.

The following validated (some adapted) outcome measures will be administered at baseline only:

#### Perceived importance of GGS

This 5-item measure adapted from Hay et al. [54] assesses perceived importance of genetic information to the participant, using a Likert scale. Specifically, the questions pertain to importance of learning about how genes affect the chance of developing cancer or other diseases, how lifestyle affects the chance of getting cancer or other diseases, and how much control the participant feels over whether they would develop cancer in the future. High scores indicate greater importance.

#### Knowledge

An 8-item, multiple choice, study-developed questionnaire assessing knowledge of the purpose of GGS, likely frequency of informative results, cancers in which informative results are more likely to be found, availability of tailored risk-management or treatment options, and source of genetic knowledge. Scores are summed, with high scores indicating greater knowledge.

#### Preferences for who should be tested

Three items to assess participants’ views on who should receive GGS if it were clinically available, for comparison with a previous study [55].

#### Value of GGS

A hypothetical time trade-off scenario based on those used in three previous studies [41, 56, 57]. Six items assess how the likelihood of finding an informative result impacts willingness to have GGS, and the amount the participant would be willing pay for GGS (from $0 to $10,000) if GGS found an informative result in 1, 10, 20, 30, 40 or 50 people out of 100.

#### Preferences for being informed of results

Four Likert-scale items adapted from Tabor et al., [58] assessing desire for results informing: treatment, prognosis, and risk of other cancers (yes / no / maybe / don’t know).

#### Self-efficacy

Four items adapted from Rosenburg et al., [59] assessing perceived ability to cope if actionable, non-actionable, incidental or germline results are found. High scores indicate greater perceived ability to cope.

#### Anticipated behavioural change

Seven Likert-scale items developed from the literature to measure the participant’s anticipated likelihood of behavioural change in the event of receiving a positive result for cancer risk. Higher scores indicate stronger intention to change behaviour. This item is included for comparison with a future study. Study-developed.

#### Tolerance for uncertainty

This measure from Kasparian et al. [60] includes 8 Likert-scale items to assess reaction to uncertainty, ambiguity and the future. High scores indicate greater intolerance.

The following outcome measures will be administered at the first and second follow-ups:

#### Perceived benefits and drawbacks of genetic panel testing

Nine Likert-scale items and two open-ended questions adapted from an earlier study [61] assessing perceived specific benefits and drawbacks of GGS.

#### Cancer specific anxiety

The 15-item Impact of Events Scale (IES) [62] assesses cancer related anxiety, in two subscales, intrusive thinking and avoidance. High scores indicate greater cancer-related anxiety.

#### Anxiety and depression

The 14-item Hospital Anxiety and Depression Scale (HADS) [63] comprises two 7-item sub-scales measuring anxiety and depression. High scores indicate greater morbidity.

#### Coping with waiting for results

Twenty-three items from the Multidimensional Impact of Cancer Risk Assessment [64] assessing impact of waiting for results of genetic testing. High scores indicate greater distress.

#### Hope

The 12-item Herth Hope Index (HHI) [65] measures hope and sense of meaning, with three subscales: temporality and future, positive readiness and expectancy, and inter-connectedness. High scores indicate greater hope.

#### Decisional regret

The 5-item Decisional Regret Scale (DRS) [66] measures health care decision regret about the decision to have GGS. High scores indicate greater regret.

The following outcome measures will be administered at baseline and all follow-ups:

#### Perceived susceptibility

Three items where participants indicate perceived likelihood of having a gene fault that increases risk of cancer occurrence or progression from ‘much lower’ (0) to much higher [4], and also on a visual analogue scale (0–100%) [60].

#### Fear of cancer occurrence/progression

The five-item Concerns about Recurrence Questionnaire (CARQ), [67] adapted to measure fear of cancer development or progression. High scores indicate greater fear.

#### Satisfaction with decision to have GGS

The 6-item Satisfaction with Decision (SWD) scale [68] measures satisfaction with decision to have GGS. Items are rated on a Likert scale. High scores indicate greater satisfaction.

### Qualitative interviews

A subset of probands (*n* = 20–40) and their participating blood relatives (*n* = 20–40) will be invited to participate in three semi-structured interviews (at baseline and each follow-up). Probands and relatives will be interviewed separately to encourage greater openness. Interviews will explore views on who should be offered GGS, attitudes to disclosure of results, perceived benefits and challenges of GGS and experiences of waiting for results. Both cohorts will be sampled purposively to include a wide variety of experiences, including individuals with and without a cancer diagnosis. Recruitment will continue until data saturation is reached.

### Qualitative data analysis

Interviews will be recorded and transcribed verbatim. Transcripts will be coded and themes will be developed through the Framework Analysis method [69]. This thematic analysis allows comprehensive and transparent data analysis within a large cohort, and for data comparisons both across and within participants, making it well suited to longitudinal data collection. Qualitative results will inform the quantitative results.

### Ethical analysis

Ethical concepts and analysis will be pertinent for the whole of this study, and will be iteratively and critically reflected upon as the project proceeds. Survey items allowing free-text responses regarding benefits and drawbacks of GGS will allow the investigating team to examine whether any responses support the identification of relevant ethical themes; such as how participants view autonomous decision-making or cost considerations. Findings from qualitative interviews will be critically compared with bioethics literature regarding concepts such as family communication, duties to disclose, what constitutes autonomous decision-making (informed consent), approaching uncertainty in GGS and determining when it is appropriate to offer testing; and to whom. The result will be a series of normative positions, supported by empirical data [70, 71].

### Quantitative data analysis

Mean differences in outcomes will be compared using t-test (continuous) or chi-squared tests (dichotomous). Non-parametric tests will also be used where appropriate. Temporal changes in scales will be investigated by calculating the difference between time-points. Multiple (continuous outcomes) or logistic (dichotomous outcomes) regression will be employed to adjust for the effect of confounders and identify predictors of outcome. The correlation structure between blood relatives or when outcomes are analysed over more than 2 time points will be suitably adjusted for when needed using mixed effects models. Linear mixed models and logistic mixed models will be performed in R: A language and environment for statistical computing employing package nlme for the linear mixed model and lme4 for the logistic mixed model. Assumptions of normality of residuals and homogeneity of variance will be confirmed visually though diagnostic residual plots. Multivariable models will be created with the inclusion of all potential confounders, and those predictors that show little evidence for an association with the outcome in univariate analysis. Backwards elimination followed by forwards addition will be employed to select predicting variables in the final model. Known and identified confounders will be incorporated regardless of their statistical significance. Collinear independent variables will be identified and eliminated.

### Sample size

The parent study plans to recruit 3000 probands in total over three years. With a conservative retention and survival estimate of 75%, this is reduced to at fewest 469 patients per cohort. With this sample size estimate, using a significance level of 0.05 this project: has 90% power to estimate the perceived value of genomic information in each time trade-off category of all patients and blood relatives to a margin of error of at most 5%; and estimate other secondary outcomes with adequate precision. Multiple regression on each cohort with at most 24 explanatory variables (including dummy variables) has 90% power to detect significant categorical variables with 5 categories (largest possible categorical variable) when that predictor explains greater than 3.6% of the residual variance, or to detect a continuous variable when it explains more than 2.3% of the residual variance.

## Discussion

The PiGeOn study will contribute to the literature by identifying the knowledge, values, attitudes and coping strategies of patients and their blood relatives with regards to GGS. The project aims to determine how these, and other factors, predict subsequent cancer-related behaviour and psychosocial outcomes (in association with a future study). This study will also describe these factors’ alignment with current ethical norms. This will be the first Australian study to collect longitudinal data on cancer patients’ experience of GGS, and one of the first studies internationally to follow probands and their blood relatives who undergo GGS, over time.

The parent study, the Cancer Risk in the Young Study, will involve participants who have the experience of a prolonged wait time until GGS results are available, and the majority of participants will receive no results (as will be explained during the consent process). Despite being advised that it is unlikely that a pathogenic gene variant will be identified, previous studies involving healthy participants suggest that patients have high expectations for the usefulness of returned results, and may therefore continue to hope for, and possibly worry about, results nonetheless [48]. The timing of the assessments allow the psychosocial impact of this wait time to be explored based on Protection Motivation Theory. Results from this study will be linked with subsequent studies to explore patient responses to results including lifestyle change and pursuing genetic counselling.

A qualitative component has been included in order to capture and explore the complexity of patient expectations, the experience of uncertainty, their understanding of the implications of testing and their attitudes towards sharing results, which may have familial repercussions. Motivation for testing, information needs and preferences are expected to vary and qualitative research has value in understanding processes.

Genomic screening, both tumour and germline, is likely to become widespread in healthcare, and to influence cancer prevention, diagnosis, treatment and risk-management. GGS generates information of unprecedented volume, some of which will have uncertain significance, the meaning of which will change over time. It gives rise to testing methods that are both diagnostic and predictive and lead to implications for communication within families and reproductive choice on a scale which we have previously never had to manage in healthcare. The study results will inform ongoing ethical debate on issues relevant to the large-scale introduction of GGS into the clinical setting, such as protocols for obtaining informed consent for GGS as well as assenting to unknown future research, and informing (or not informing) patients and their relatives regarding genomic results. The study will also assess whether there are psychosocial sequelae following GGS testing, after being informed that results for patients and their blood relatives may not be forthcoming. Results will be linked to further studies to inform understanding of behaviour following receipt of results. These data are needed to ensure that when GGS is commenced as part of routine clinical care, ethical principles are well-established in practice, and patients and their relatives receive sufficient preparation and support before, during and after GGS testing. While this cohort is a selective sample with risk factors suggestive of a germline mutation such as young age of onset or multiple primaries, understanding the influences on behavioral change will also increase the likelihood that the introduction of germline genomic cancer screening for the general population will have the hoped-for positive benefits.

Given the scarcity of evidence on responses to actual GGS for cancer, this study is an important and timely step in filling the critical gaps in understanding about the best way to introduce GGS into clinical medicine.

## Abbreviations

DNA: Deoxyribonucleic acid
GGS: Germline genome sequencing
SD: Standard deviation
USA: United States of America
